# Aesthetic Preference for Negatively-Valenced Artworks Remains Stable in Pathological Aging: A Comparison Between Cognitively Impaired Patients With Alzheimer's Disease and Healthy Controls

**DOI:** 10.3389/fpsyg.2022.879833

**Published:** 2022-05-26

**Authors:** Elisabeth Kliem, Michael Forster, Helmut Leder

**Affiliations:** ^1^Department of Psychology, Norwegian University of Science and Technology, Trondheim, Norway; ^2^Faculty of Psychology, University of Vienna, Vienna, Austria

**Keywords:** Alzheimer's disease, aesthetic preference, emotional valence, recognition memory, preference stability, neurodegenerative disorder, dementia

## Abstract

**Background:**

Despite severe cognitive dysfunction in Alzheimer's disease (AD), aesthetic preferences in AD patients seem to retain some stability over time, similarly to healthy controls. However, the underlying mechanisms of aesthetic preference stability in AD remain unclear. We therefore aimed to study the role of emotional valence of stimuli for stability of aesthetic preferences in patients with AD compared to cognitively unimpaired elderly adults.

**Methods:**

Fifteen AD patients (Mini-Mental State Examination (MMSE) score 12–26) without visual impairment and/or psychiatric disorder, as well as 15 healthy controls without cognitive impairment (MMSE ≥ 27) matched in age, sex, art interest and highest level of education were included in this study. All participants were asked to rank-order eight artworks per stimulus category (positive, negative, neutral in emotional valence) according to their preference twice with a 2-week span in-between. Based on these two rankings a preference change score was calculated. In order to assess explicit recognition memory of the artworks in the second testing session, four artworks of each stimulus category used in the preference ranking task were presented together with a content-matched distractor artwork painted by the same artist. Participants had to indicate which of the stimuli they had seen 2 weeks previously.

**Results:**

AD patients [MMSE (*M*) = 18.9 ± 3.6; Age (*M*) = 85.4 ± 6.9; 33.3% male] had no explicit recognition memory of the artworks (recognition at chance level), whereas healthy controls [MMSE (*M*) = 27.7 ± 1.4; Age (*M*) = 84.3 ± 6.7; 33.3% male] correctly recognized 85% of stimuli after 2 weeks. AD patients had equally stable preferences compared to the control group for negative artworks, but less stable preferences for positive and neutral images (Bonferroni-corrected significance levels; *p* < 0.017).

**Conclusion:**

Even in cognitively impaired AD patients, aesthetic preference for negatively-valenced artworks remains relatively stable. Our study provides novel evidence that AD patients may have a somewhat preserved implicit valence system for negative compared to neutral or positive visual information, especially in the domain of aesthetics. However, more studies need to further uncover the details of the underlying neurocognitive mechanisms of preference stability in pathological aging.

## Introduction

Alzheimer's disease (AD) is a neurodegenerative disorder and the most common cause of dementia, doubling in prevalence every 5 years after age 65 (Lane et al., 2018). Besides the societal and economic impacts following these developments, this disease poses severe physical and psychological challenges to the suffering individual: With its progressive pathological aging of the brain, AD leads to different clinical symptoms including cognitive, emotional and behavioral dysfunction, failure to maintain activities of daily living (Scheltens et al., 2016) and changes of personality (Chatterjee et al., 1992; Balsis et al., 2005). Significant memory impairment, especially in long-term declarative memory, is one of the early symptoms and core characteristics in AD (Jahn, 2013), and patients often gradually experience social isolation and decreased quality of life when the disease progresses.

For AD patients, engagement with art, like visiting a gallery, seems to be a promising approach to enhance social interactions and to increase quality of life (Flatt et al., 2015). Such aesthetic experiences are marked by strong interactions between cognitive and emotional pathways (Leder and Nadal, 2014). When engaging with art, these interactions may differ from experiences in other settings as negative emotions elicited by the artwork may contribute to a pleasurable experience (Menninghaus et al., 2017), making art an interesting avenue to study emotional evaluations and their interaction with higher-order cognitive processes.

One of the underlying reasons why engagement with art has beneficial effects for AD patients might be that the very ability to form and retain a subjective aesthetic judgment seems to be preserved. More specifically, aesthetic preference ratings of individuals with dementia seem to be no less stable over time than those of healthy controls (Halpern et al., 2008; Graham et al., 2013; Halpern and O'Connor, 2013; Pugach et al., 2017): When participants had to rank-order images depicting representative, quasi-representative and abstract art according to their subjective liking twice with a 2-week span in-between, similar degrees of preference stability in healthy participants and AD patients have been found, even though AD patients had poor explicit memory of the artworks (Halpern et al., 2008). Similarly, stability of preferences in AD patients was similar to those of controls for portraits, landscape paintings and landscape photographs, but not for photographs of faces (Graham et al., 2013). These findings have also been reported in patients with frontotemporal dementia (Halpern and O'Connor, 2013), and have been replicated in a study with higher ecological validity using artworks displayed in a gallery (Silveri et al., 2015).

Yet, the underlying mechanisms of retained stability of aesthetic preferences in dementia remain somewhat unclear: Art style does not seem to be a primary factor in generating stability (Halpern et al., 2008; Silveri et al., 2015), and mixed results have been found about the role of the developmental stage of AD (Halpern et al., 2008; Graham et al., 2013). Also, with explicit memory for artworks being poor in dementia patients, benefits due to recognition of artworks as a cause for retained stability are unlikely.

Given the rich interplay of emotional and cognitive processes in aesthetic appreciation (Leder and Nadal, 2014), studying the effect of stimulus valence might shed some light on the potential causes of the preserved stability. This might be a promising field of study even in the cognitively impaired, because AD patients have shown to retain the ability to form correct judgments of the valence and arousal aspects of emotions (Burton and Kaszniak, 2006; Henry et al., 2009), to correctly differentiate between emotions with different valence (Bucks and Radford, 2004), and also to form aesthetic judgments that correspond with the artworks' valence *(ugly/unpleasant; beautiful/pleasant)* similar to healthy controls (Boutoleau-Bretonnière et al., 2016). In addition, emotional valence of stimuli seems to be implicitly retained in AD patients even when explicit memory of these stimuli is lost (Guzmán-Vélez et al., 2014), which indicates that acquisition and maintenance of implicit affective dispositions may be preserved in in this disease (Blessing et al., 2006, 2010). Moreover, emotionally-valenced information has shown to enhance attention and memory compared to neutral stimuli both in healthy individuals (Murphy and Isaacowitz, 2008), and, to some extent, in cognitively-impaired AD patients (Broster et al., 2012).

Taken together, AD patients seem to maintain the ability to experience emotions through visual stimuli and to correctly judge their valence, as well as to benefit to some degree from emotional valence for attentional and memory processes. Based on these findings, this study now aimed to explore the role of emotional (i.e., positive and negative) valence of artworks for the stability of preferences in AD patients compared to an age-, sex-, art interest-, and education-matched healthy control group over a 2-week period. Specifically, we first intended to explore whether recognition memory of artworks differed between healthy individuals and AD patients, and whether memory performance was influenced by valence of the stimuli. Second, we aimed to explore whether valence of stimuli impacted aesthetic preference stability, and whether these effects differed between groups.

Concerning recognition memory, we hypothesized that individuals with AD will remember fewer artworks of all stimulus categories (positive, negative, neutral) compared to controls. With previous research indicating emotional enhancement effects on cognitive function, we furthermore hypothesized that healthy individuals remember significantly more positive and negative compared to neutral stimuli, whereas we did not expect such emotional enhancement effects in the AD group.

Based on findings which showed a facilitation of implicit cognitive processes due to emotional content, also in the cognitively impaired, we furthermore predicted higher stability of preference for artworks with positive or negative valence compared to neutral artworks in both groups. Finally, possible confounding factors such as severity of general cognitive impairment, arousal of stimuli, participants' mood and the consistency of ranking positions for each individual stimulus were analyzed *post-hoc*.

## Methods

The study received ethical clearance from the Ethics Committee of the University of Vienna (Reference Number: 002800, 11/28/2017) and was online pre-registered at Aspredicted.org (Reference Number: 14229, 09/20/2018; https://aspredicted.org/blind.php?x=3cc55q). All participants gave their informed consent according to the Declaration of Helsinki and did not receive any incentives for taking part in this study.

### Study Design

Participants were told to take part in a study on “Art appreciation in elderly people” and were unaware of the purpose of the study. All participants were tested in the morning between 9 a.m. and 1 p.m., and all tests were performed in German language by the same researcher (EK) in order to enhance comparability between results.

The present study consisted of two testing-sessions with a 2-week span in-between, following a similar procedure as previous studies on aesthetic stability in AD patients (Halpern et al., 2008; Graham et al., 2013). Both sessions started with a visual control task in which participants were asked to rank-order colored drawings of eight everyday objects (Rossion and Pourtois, 2004) according to real-world size. The aim of the control task was to test for visual impairment as well as to test whether participants were able to understand and correctly carry out verbal instructions. As we observed difficulties among dementia patients to conduct a free ranking task in a pre-study (*n* = 3 per group), we changed the task for the main study into a forced-choice format so that only two stimuli at a time were displayed. The stimuli included in this study were paired as follows: balloon (*no. 015*)—button (*no. 041*), horse (*no. 121*)—airplane (*no. 002*), apple (*no. 006*)—elephant (*no. 084)*, chair (*no. 053*)—house (*no. 122*). The numbers refer to the stimulus number from the original image-set by Rossion and Pourtois (2004).

After that followed the preference ranking task, in which participants were asked to rank-order eight artworks for each of the three stimulus sets (positive, neutral, negative) according to their aesthetic preference. One stimulus set at a time, all eight stimuli were displayed on a table in two rows in random order. The order of stimulus sets itself was counterbalanced across groups, so that all possible sequences were equally often shown in the AD and control group. Participants were asked to spatially sort the artworks from left to right according to their aesthetic preference in descending order. In the AD group, instructions were repeated multiple times throughout the task in order to make sure that participants correctly remembered instructions. There was no time limit for the ranking task and positions could be changed during the session. Participants were asked to only judge according to their own aesthetic preference and told there were no correct or wrong “answers”.

The second session followed the same procedure as the first, but included an additional recognition memory task which was administered before the preference ranking task. The aim of this task was to make sure that participants in the control group remembered the stimuli they had seen 2 weeks previously and to confirm that this was not the case for AD patients. In this task, four pairs of images per stimulus set, each comprising one image from the first testing session plus a distractor image, were laid out in randomized order in front of the participants. Participants had to indicate which of the images they had seen 2 weeks previously and the number of correct answers was measured.

### Participants

#### AD Patients

AD patients were recruited *via* two nursing homes in Germany and *via* a daily care center for people with dementia in Austria. All participants included in the patient group had a current diagnosis of AD as diagnosed by authorized clinicians. Only AD patients with a Mini-Mental State Examination score (MMSE) (Folstein et al., 1975) ≥ 12 were included in the study as we assumed that more severe cognitive impairment would lead to difficulties in understanding and conducting our tasks. In addition, patients showing one or more of the following criteria were excluded from the study: (1) current or previous self-reported diagnosis of any psychiatric disorder, including depression: Individuals with depression show preferential attention to negatively-valenced stimuli and might encode valence differently from individuals without a history of depression (Kerestes et al., 2012; Liu et al., 2012); (2) high art expertise: Art experts were excluded from this study, because they exhibit different visual processing of emotionally-valenced art compared to lay people (Leder et al., 2014); (3) early-onset AD: Individuals with early-onset AD show a different course of disease and cognitive profile compared to late-onset dementia (Smits et al., 2012; Joubert et al., 2016). Our minimum age for inclusion in this study was therefore set to 65 years. Finally, (4) individuals with perceptive impairments that might impact the visual processing of the artworks were excluded. This was tested with a specific control task (see section Study Design).

From an initial set of 21 participants, four participants were excluded because they could not finish the second test session due to illness, one participant was excluded because of missing values in the preference ranking tasks and one participant was retrospectively excluded due to a diagnosis of schizophrenia of which the authors were not aware of at the time of testing. Therefore, data of 15 AD patients were included in the final analyses which is similar to the sample size of previous studies on preference stability in dementia (Halpern et al., 2008; Graham et al., 2013).

#### Control Group

Control participants were recruited *via* the same nursing homes as AD patients in Germany and Austria, as well as among family members/caregivers of AD patients, and *via* the social network of the researchers. Only people with MMSE-score ≥ 27, without known history of dementia, depression or any other psychiatric disorder (based on self-report), without perceptual impairments (based on the control task) and who were no art experts (based on self-report) were included in the control group. Each participant in the control group was exactly matched to one AD patient according to age (±3 years) as well as sex. In many cases, we also found exactly matching pairs for self-rated art interest and highest level of completed education. In some cases, where it was difficult to find exactly matching pairs, AD patients and healthy controls were matched with ± one level difference in art interest and education. From an initial set of 16 control participants, one person did not take part in the second testing session due to illness and was therefore excluded. Thus, data of 15 controls were included in the analyses of the study, leading to 15 matched pairs.

### Stimuli

All images (preference ranking and recognition task) were printed in color. They had a size of 18 × 13 cm, with a white frame of at least 0.5 cm, depending on the format of the original artworks. A complete stimulus list is shown in Table 1.

**Table 1:** List of stimuli used in the preference ranking and the recognition memory task.

**Stimulus category**	**Artist**	**Painting**	**Distractor**
**Negative**			
NG1	Ernst	The anti-pope	
NG2	Ensor	The intrigue	Masks confronting death
NG3	Masson	Tauromachie	
NG4	Munch	Ashes	Separation
NG5	Lassnig	Three ways of being	
NG6	Van Honthorst	Susanna and the elders	The steadfast philosopher
NG7	Brugghen	David praised by the Israelite women	
NG8	Dix	Skat players	Prager street
**Neutral**			
NT1	Crespi	Christ and the Samaritan woman	
NT2	Wyeth	Christina's world	Winter 1946
NT3	Bernard	Breton women at a wall	
NT4	Reynolds	George Clive and his family with an Indian maid	Lady Cockburn and her three eldest sons
NT5	Leibl	Three women in church	
NT6	Balthus	The street	The passage of Commerce Saint-Andre
NT7	Hopper	Room in New York	
NT8	Vermeer	Woman reading a letter	Girl reading a letter at an open window
**Positive**			
PO1	Slevogt	The dancer Marietta di Rigardo	Anna Pawlowa
PO2	Boucher	The bird catchers	
PO3	Wright of Derby	Three persons viewing the gladiator by candlelight	
PO4	Vallotton	The visit	The lie
PO5	Renoir	Dance at the Moulin de la Galette	
PO6	Gaugin	Nave Nave Mahana	
PO7	Cassatt	The child's bath	Emmie and her Child
PO8	Manet	Argenteuil	Boating

*When a distractor image is listed, the artwork was included in the recognition memory task*.

#### Preference Ranking Task

Eight artworks for each of the three stimulus sets (positive, negative, neutral) were selected from the *Vienna Art Picture System* (Fekete et al., 2022) which contains a total of 1,000 artworks in five categories (scenes, portrait, landscape, still life, abstract art). Different art styles were included, because previous studies did not find an effect of art style on preference stability in AD and their controls (Halpern et al., 2008; Silveri et al., 2015), but only artworks from the subcategory *scenes* were chosen, so that each image depicted one or more figures interacting with their environment. All stimuli in the database are pre-rated on several dimensions including emotional valence on a 7-point Likert scale (1 = *very negative* to 7 = *very positive*). The mean valence ratings in the subcategory *scenes* range from a minimum of *M* = 1.60, *SD* = 0.88, to a maximum mean valence of *M* = 5.25, *SD* = 1.21. The positively-valenced stimulus set had a mean valence rating from *M* = 4.30, *SD* = 1.26 to *M* = 5.25, *SD* = 0.85, the neutral stimulus set from *M* = 3.40, *SD* = 0.82, to *M* = 3.80, *SD* = 1.15, and the negative stimulus set from *M* = 2.60, *SD* = 0.99 to *M* = 2.80, *SD* = 1.44.

None of our stimuli included portraits/close-up depictions of faces, due to faces being processed differently than other objects (Bruce and Young, 1998), and their aesthetic evaluation being a specific class of aesthetic judgments (Fletcher-Watson et al., 2008). Only artworks with mean familiarity rating lower than an average of 3.5 (from 1 = *unknown* to 7 = *very familiar*) were included in order avoid possible biases on recognition and preference ranking task due to previous familiarity with the artworks (*mere exposure effect)* (Zajonc, 1968). None of the artworks included explicit scenes of death, crime, or depictions of blood in order to prevent too much emotional distress in participants.

#### Recognition Task

Twelve distractor images (four per stimulus category) were used for the recognition task. Distractor images were painted by the same artist as the original test stimulus, were matched in content and displayed similar objects or figures (see Table 1).

### Measures

To measure preference stability, we first calculated a preference change score using the same method as applied in previous studies (Halpern et al., 2008; Graham et al., 2013). Therefore, we first counted the change in positions that each item had in the preference ranking of Session 1 compared to Session 2. For example, if Picture A was ranked on the first position in Session 1 and on the last position (Position 8) in Session 2, it changed in rank by seven positions. Added up, the position changes of all items in one stimulus category generated a total change score, which was then divided by eight, the total number of items. This resulted in a mean change score indicating how many ranks (on average) images of each category changed between sessions. Final change scores could range between 0 and 4, with 0 indicating a perfect stability between the two ratings (i.e., all pictures were rated in the same order) and with a value of 4 indicating a maximal low consistency between the two ratings. Thus, *higher change scores indicate lower preference stability*. These change scores were calculated for each valence category separately.

In addition, age, sex, general cognitive function, highest level of completed education, mood and art interest were assessed. Art interest was measured on a 7-point scale ranging from 1 (=*no interest at all*) to 7 (=*very high interest*). Highest completed level of education was measured with four clusters representing the German and Austrian school system (1 = *Volksschule (i.e., 5–7 years of education)*, 2 = *Hauptschule/ Mittelschule/Realschule (i.e., 9–10 years of education)*, 3 = *Gymnasium/weiterführende höhere Schule (i.e., 13 years of education)*, 4 = *Universitätsstudium, (i.e.*, >*13 years of education*). For exploratory analyses we also measured current mood of participants in the beginning of both sessions, by asking the question “How are you feeling today?” with a 5-point scale from 1 (= *very bad*) to 5 (= *very good*). Following the suggestion of Stern et al. (1997) to use visual analog scales for cognitively impaired individuals when assessing internal emotional experiences, we used five small icons (“Smileys”) together with the scale to measure mood in the AD group.

To measure general cognitive function, all participants were tested with the MMSE before the first testing session. Total scores range from 0 to 30, with higher scores indicating better general cognitive function.

## Results

All statistical analyses were conducted with the R Statistical Computing Software (https://www.R-project.org/), version 3.5.1 (07/02/2018). For all analyses, Bonferroni corrections were used to adjust for multiple comparisons. To estimate effect size, we used partial eta-square (${\text{η}}_{\text{p}}^{2}$) for analyses of variances (*ANOVA*) and Cohen's *d* for *t*-tests. Cohen's (1988) classification was used for ${\text{η}}_{\text{p}}^{2}$ where values ≥0.01 are interpreted as small, ≥0.06 as medium and ≥0.14 as large effect. For *t*-tests, a Cohen's *d* ≥0.20 indicates a small effect, *d* ≥0.05 a medium effect and *d* ≥0.80 a large effect (Cohen, 1988).

### Sample Characteristics

Sample characteristics are displayed in Table 2. All participants were Caucasian, and native German speakers. AD patients showed on average moderate levels of cognitive impairment, with scores ranging from 12 to 26. Mood was stable between Session 1 and Session 2 in both groups, but significantly lower in the AD group compared to controls in both sessions [Session 1: *t*_(28)_ = −2.80, *p* = 0.009, *d* = 1.02; Session 2: *t*_(28)_ = −3.54, *p* = 0.001, *d* = 1.29].

**Table 2:** Sample characteristics.

	**AD group (*n* = 15)**	**Control group (*n* = 15)**
Age 69–97 (SD), y	85.40 (6.9)	84.33 (6.7)
Education (SD), range 1–4	2.13 (1.1)	2.27 (1.2)
Sex, *n* male (%)	5 (33.3%)	5 (33.3%)
Art interest, mean (SD), range 1–7	4.47 (1.2)	4.47 (1.4)
MMSE-score, mean (SD)	18.87 (3.6)	27.73 (1.4)
Mood session 1, mean (SD), range 1–5	3.20 (1.1)	4.20 (0.9)
Mood session 2, mean (SD), range 1–5	3.20 (0.9)	4.20 (0.7)

### Visual Control Task

The majority of all participants was able to conduct the task correctly in both sessions: As expected, in the control group the answer rate of correct responses was 100% in both sessions. In the AD group, 73% (*n* = 11) answered all items correctly and another 20% (*n* = 3) made one mistake in the first testing session. Only one participant made two errors. In the second testing session, AD patients showed very similar results, with 66% (*n* = 10) answering everything correctly and four participants (27%) answering three out of four correctly. Again, only one participant had half of the forced-choice items correct. Furthermore, the two lowest scores stemmed from two different persons in both sessions who had low MMSE-scores (12 and 14, respectively), and thus, general cognitive impairment rather than visual impairment may have contributed to low scores in the control task. However, all participants of both groups and in both sessions were able to name the depicted object correctly.

Even though a few of the participants in the AD group had trouble answering all items correctly, compared to the results of Graham et al. (2013) who used the more complicated free ranking task, our participants had much less difficulties in the control task in general, and none of them scored very badly in this task or were completely unable to conduct it. With roughly 93% of AD patients showing only few difficulties with this task, we concluded that the majority of AD patients did not show any visual impairments that would interfere with our tasks. In order to make sure that patients with more severe cognitive impairment and difficulties with the visual control task would understand and perform our instructions correctly, we repeated instructions multiple times throughout the following recognition and preference ranking tasks.

### Recognition Task

The overall mean of correct recognitions in the control group was 3.38 (*SD* = 0.72) out of four, whereas the mean of correct choices in the AD group were at chance level (*M* = 1.98, *SD* = 0.78). Thus, controls recognized 85%, while AD patients only recognized 50%. Furthermore, the mean of correct responses for each stimulus category (i.e., negative, positive, and neutral images) was also roughly at chance level in the AD group. The means of all recognition scores and standard deviations for all stimulus categories and both groups are depicted in Table 3.

**Table 3:** Means and SDs of recognition scores per stimulus category and group.

	**Negative *M (SD)***	**Neutral *M (SD)***	**Positive *M (SD)***
AD	2.00 (0.85)	2.13 (0.64)	1.80 (0.86)
Controls	3.27 (0.46)	3.40 (0.83)	3.47 (0.83)

*Higher recognition scores indicate better memory*.

To test our hypothesized main effect for group differences between AD patients and controls in recognition scores, we conducted a repeated-measures ANOVA with correct recognition scores as the dependent variable and stimulus category (positive/negative/neutral) as well as matched group (AD/control) as within-participants factors. As predicted, the results showed a strong main effect of group *F*_(1, 14)_ = 101.77, *p* < 0.001, η^2^_p_ = 0.88. For the main effect of valence of stimulus category, the assumption of sphericity was not met (*p* = 0.009), and Greenhouse-Geisser corrections were used. There was no main effect of valence of stimulus category, *F*_(1.32, 18.45)_ = 0.40, *p* = 0.589, η^2^_p_ = 0.03. Also, there was no interaction effect between group and stimulus category, *F*_(2, 28)_ = 0.46, *p* = 0.638, η^2^_p_ = 0.03. This shows that controls recognized significantly more images of all stimulus categories than AD patients. Furthermore, controls did not recognize more pictures with positive or negative valence compared to neutral stimuli. Instead, controls showed equally well performance in all stimulus categories, *F*_(2, 28)_ = 0.30, *p* = 0.741, η^2^_p_ = 0.02.

Even though mean recognition in all stimulus categories was around 50% in the AD group, a few participants showed very good performance in the recognition memory task (Supplementary Figure 1). Descriptive analyses showed that each of these outliers stemmed from different participants, thus no participant scored very high (i.e., three or four correct) in two or all three stimulus categories in one session. In order to confirm that these outliers were accumulated by chance, we compared the mean of correct recognition rates of each stimulus category to correct recognition scores at chance level using one sample *t*-tests. The mean recognition performance did not significantly differ from recognition rates at chance level in every stimulus category [positive category, *t*_(14)_ = −0.90, *p* = 0.384, *d* = −0.23; negative category, *t*_(14)_ = 0.00, *p* > 0.999, *d* < 0.01; neutral stimulus category, *t*_(14)_ = 0.81, *p* = 0.433, *d* = 0.21]. Furthermore, we compared total recognition memory (i.e., recognition memory for all pictures of all stimulus categories) between controls and AD patients (Supplementary Figure 2). In total, the control group had 152 hits and 28 false alarms, and the AD group 89 hits and 91 false alarms. The standardized difference between hit and false alarm rates (*d*-prime, *d*′) was 2.03 for the control and −0.03 for the AD group. *D*-prime was significantly different from 0 for the control group [*t*_(14)_ = 5.07, *p* < 0.001, *d* = 1.31] and not significantly different from 0 in the AD group [*t*_(14)_ = −0.28, *p* = 0.787, *d* = −0.07], which indicates performance on chance level for recognition memory in the AD group.

### Preference Ranking Task

**Figure 1:**
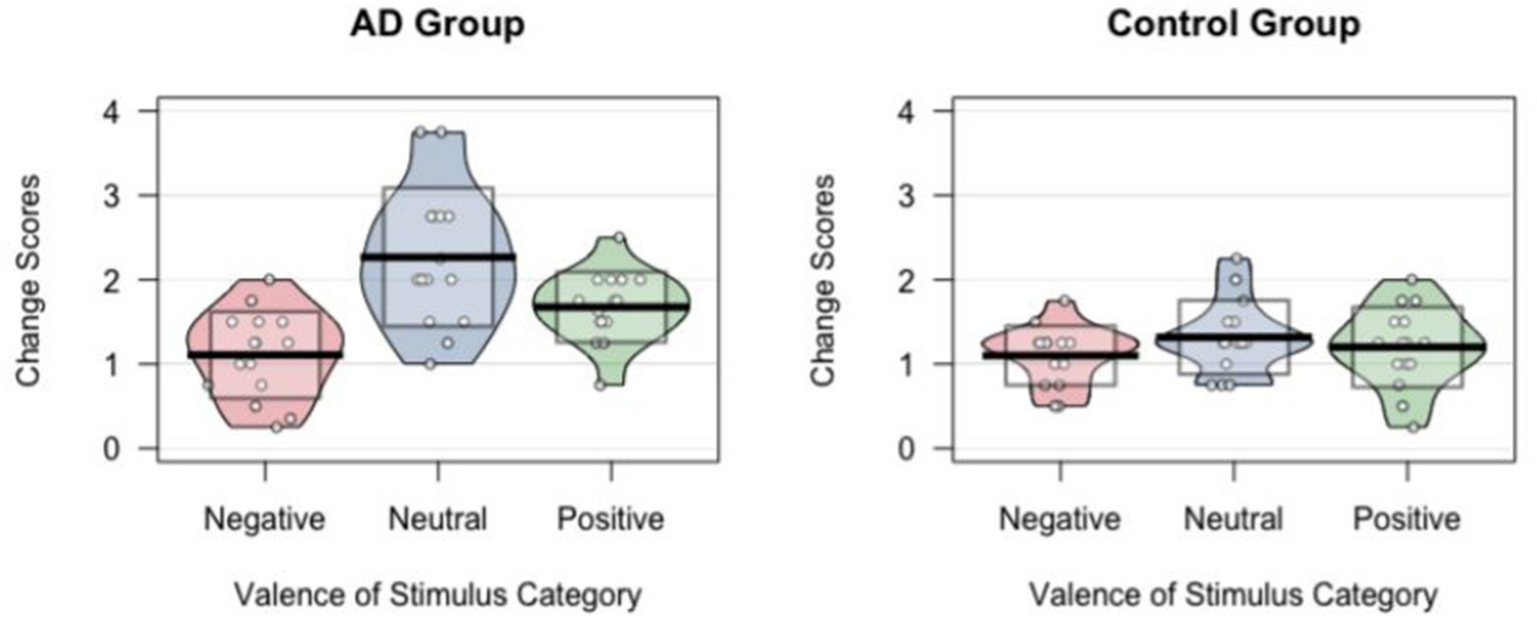
Distributions of preference change scores shown per stimulus category and group. Each dot represents the preference change score for one participant. Noise (jitter) was added horizontally to reduce overlap among points with similar value. Horizontal black lines indicate means. Standard deviation of the mean of preference change scores for each valence category are indicated as transparent boxes.

Table 4 shows the mean change scores (and their *SD*s) in all three stimulus categories and for both groups. A distribution of all mean change scores grouped by stimulus category and group are shown in Figure 1.

**Table 4:** Means and SDs of change scores per stimulus category and group.

	**Negative *M (SD)***	**Neutral *M (SD)***	**Positive *M (SD)***
AD	1.11 (0.51)	2.27^*^ (0.82)	1.68^*^ (0.42)
Controls	1.10 (0.35)	1.32 (0.44)	1.20 (0.47)

* *Significant at p < 0.017 (Bonferroni correction)*.

*Lower mean change scores indicate higher stability*.

A repeated-measures ANOVA with preference change scores as the dependent variable and stimulus category (positive/negative/neutral) as well as matched group (AD/control) as the within-participants factors showed a large main effect of valence of stimulus category [*F*_(2, 18)_ = 11.63, *p* < 0.001, η^2^_p_ = 0.45] and of group [*F*_(1, 14)_ = 26.06, *p* < 0.001, η^2^_p_ = 0.65] as well as a significant interaction effect [*F*_(2, 28)_ = 5.21, *p* = 0.012, η^2^_p_ = 0.27].

The significant interaction effect was further analyzed with *post-hoc* comparisons using *t*-tests with Bonferroni correction. With a critical alpha-level of 0.017 (Bonferroni correction: 0.05/3), a significant difference between the AD and the control group was found for neutral, *t*_(14)_ = 3.85, *p* = 0.002, *d* = 0.99, and positive images, *t*_(14)_ = 2.97, *p* = 0.010, *d* = 0.77, with the AD group showing significantly less stability. No significant group difference was found for negative stimuli, *t*_(14)_ < 0.01, *p* > 0.999, *d* < 0.01.

We also studied differences in change scores between stimulus categories *within* groups using *t*-tests with Bonferroni correction (by multiplying *p*-values by the number of comparisons using the *p.adjust* function in *R*) for pairwise comparisons. Our analyses for the AD group revealed significant differences between positive and negative (*p* = 0.043, *d* = 1.22), between positive and neutral (*p* = 0.033, *d* = 0.91), as well as between neutral and negative (*p* < 0.001, *d* = 1.70). In the control group, no significant differences between any stimulus categories were found (all *p*s ≥ 0.51, all *d*s ≤ 0.24).

### *Post-hoc* Tests to Study Possible Confounding Variables

#### Severity of Cognitive Impairment

We investigated a possible relationship between aesthetic stability and severity of general cognitive impairment as previous studies showed mixed results (modest correlation for quasi-representational artworks in Halpern et al., 2008; no correlation Graham et al., 2013).

Pearson correlation showed no significant relationship between MMSE-score and preference change score for negative, neutral, or positive images (Table 5).

**Table 5:** Relationship between severity of cognitive impairment and preference change score using Pearson correlation.

	**Change score negative**				**Change score neutral**				**Change score positive**			
		**95%CI**				**95%CI**				**95%CI**		
	***r***	**Lower**	**Upper**	***p***	***r***	**Lower**	**Upper**	***p***	***r***	**Lower**	**Upper**	***p***
MMSE	0.52	0.006	0.814	0.049	0.14	−0.398	0.611	0.609	−0.57	−0.838	−0.083	0.026

*Significance level p < 0.017 (Bonferroni correction)*.

#### Item Consistency

As it might be possible that stable preference rankings are based on the fact that some artworks were generally preferred or disliked, we analyzed the consistency of ranking positions for each individual stimulus and for the AD and control group separately. In order to see how much an artwork was generally liked (indicated by a low average ranking) or disliked (indicated by a high mean ranking), we calculated the average ranking for each artwork in each stimulus category at the first testing session. The mean average rankings per stimulus over all categories ranged from 2.14 to 6.40 in the control group. In the AD group the range of average rankings for artworks was a little bit wider, with means from 1.94 to 6.80.

We followed the procedure of Halpern et al. (2008) and had a look at those paintings at the “endpoints of rankings”, which were defined as one rank below and above the median on a 1–8 scale (4.5), i.e., images with a mean ranking below 3.5 and over 5.5. In general, our results on artworks at the endpoints of rankings are similar to Halpern et al. (2008, Experiment 2).

Pictures that were generally more liked among participants (i.e., a mean average rank below 3.5) were equally distributed in all stimulus categories (positive, negative, neutral) and in both groups (AD, control), with one such image in each stimulus category for both AD patients and controls. This makes it rather unlikely that there was a universal preference of images in one particular stimulus category which could have influenced the results. For rather disliked images, three images had scores above 5.5 (all of the negative category) in the AD group, and in the control group seven images were above that threshold (two positive, two neutral and three negative stimuli). Despite these differences, even in the control group only 17% (7 images out of 24) were generally rather disliked, which we consider as rather low. General agreement on artworks was overall higher in the control group than in the AD group (10 out of 24 “extreme range images” in the control group and six out of 24 in the AD group).

To better understand the variability between ratings (i.e., disagreement between rankings), we had a closer look at the standard deviations for each stimulus and per group. The range of all standard deviations for every individual stimulus varied from 1.29 to 3.03 in the control group and from 0.96 to 2.49 in the AD group, indicating slightly less disagreement in the AD group. However, on average all *SD*s for all stimulus categories were roughly at 2 for both groups which indicates a relatively large disagreement over stimuli in both groups and matches the findings in the Halpern et al. (2008) study.

Also, in the AD group, only two stimuli (out of 24) did not have a rank range from Position 1 or 2 to Position 7 or 8. In all the other cases, at least one person ranked the painting as most or 2nd most preferred, whereas at least one person ranked the same stimulus as most or 2nd most disliked. In the control group, a total of four images (out of 24) showed this pattern. Taken together, in only six cases (out of 48) was disagreement between participants not as pronounced as with the majority of stimuli, which is in accordance with previous findings (Halpern et al., 2008).

#### Arousal of Stimuli

As arousal of our stimuli might also have influenced stability of preferences, we compared arousal values for all stimulus categories as rated in *VAPS*. Emotional arousal was measured on a 7-point scale (1 = *very calm*, 7 = *very excited*) to “Looking at this artwork makes me feel …”. Despite their differences in valence, the arousal means for positive stimuli (*M* = 3.78, *SD* = 1.21) and for neutral images (*M* = 3.80, *SD* = 1.29) were very similar, whereas negative images had higher levels of arousal, *M* = 4.85, *SD* =1.05. However, differences among arousal rankings did not lead to a generally higher or lower preference of specific artworks in this sample. The standard deviations of preference rankings for the two images per stimulus category with highest arousal ratings (*PO1, PO5; NG5, NG8*; *NT2, NT4)* were around 2–3, indicating a fairly large disagreement in preferences between participants.

#### Mood

We also studied possible mood effects on recognition rate and preference stability using mixed ANOVAs. There was no effect of mood on either recognition rate or change score in both groups and in all stimulus categories. All results are displayed in Table 6.

**Table 6:** Effects of mood on recognition and preference task.

	***df_***Num***_***	***df_***Den***_***	***F***	***p***	** ${\text{η}}_{\text{p}}^{\text{2}}$ **
**Recognition task**				
AD Mood 1	2	12	0.10	0.904	0.02
AD Mood 2	1	13	0.04	0.843	<0.01
Control Mood 1	1	13	0.04	0.843	<0.01
Control Mood 2	1	13	0.02	0.883	<0.01
**Preference task**				
AD Mood 1	1	2	0.55	0.593	0.08
AD Mood 2	2	12	0.60	0.562	0.09
Control Mood 1	1	13	0.00	0.971	<0.01
Control Mood 2	1	13	0.12	0.736	<0.01

*Mood 1 = Mood measured at 1st testing session, Mood 2 = Mood measured at 2nd testing session*.

## Discussion

The aim of this study was to explore the effects of emotional valence of stimuli on the stability of aesthetic preferences in patients with Alzheimer's dementia and healthy controls. This extends previous research on aesthetic preference stability in AD and is a crucial step to shed further light on aesthetic processing in pathological aging. Our results indicate that even in cognitively impaired AD patients without explicit recognition memory of artworks, aesthetic preference for negatively-valenced artworks remains relatively stable. Thus, this study provides a first glimpse into the role of affective valence of stimuli for aesthetic stability and may indicate that AD patients may have a somewhat preserved implicit valence system for negative compared to neutral or positive visual information, especially in the domain of aesthetics.

One possible explanation might be that this intact implicit retention of negative valence is one of the most basic processes in emotional and cognitive function that has been preserved, even in the cognitive impaired. This is in line with previous studies showing that implicit emotional learning in AD patients seems to be intact and more pronounced for negative material (sad vs. happy, Guzmán-Vélez et al., 2014). In addition, negative images may be more distinct than positive or neutral images, and may have been perceived more intensely in the AD group in general: Research on the intensity of aesthetic judgments showed that the perception of paintings as ugly and unpleasant lead to higher intensities of aesthetic judgment compared to stimuli perceived as more beautiful and pleasant only in persons with dementia, but not in controls (Boutoleau-Bretonnière et al., 2016). However, the details of the underlying neurocognitive mechanisms of preference stability in pathological aging are still poorly understood, specifically in the domain of aesthetics.

Moreover, preference for positive and neutral pictures was not equally stable in both groups, with AD patients having significantly lower stability for these images compared to healthy controls. One possible reason may be that we were restricted in the choice of artworks for this study due to exclusion of artworks with depictions of faces/portraits or with high familiarity, and thus, even our image with the highest positive valence value (valence rating of 5.25 out of 7), was not extremely positive. This may have led to a rather vague discrimination between positive and neutral stimuli in general. However, preferences for both negative and positive material was more stable in the AD group than preferences for neutral images.

It is interesting that our findings on the stability of aesthetic preference differ from previous studies that showed relatively stable aesthetic preferences in persons with dementia compared to controls *across stimulus sets*. One possible reason for this is that the artworks used in this study might have been differently processed between healthy controls and AD patients. Overall, AD patients seemed to “take in” the emotional valence conveyed by the pictures more thoroughly than controls. Being cognitively impaired, AD patients might not have abstracted as much as controls that the images were not showing real-life scenes but artworks. For example, some AD patients asked if the artwork shown was a photograph of them in their childhood or of a certain event that happened earlier in their life. In contrast, knowing that they were confronted with artworks, controls seemed to distance themselves from the images more than AD patients did. This may have resulted in a “filtered” encoding with less emotional weight attached to it. This is in accordance with empirical evidence that suggests that framing an image as “art” changes cognitive processes in the viewer: Negative images that were believed to be artworks were generally more appreciated than when participants believed they were real-world scenes (Wagner et al., 2014), which was also mirrored by changes in psychophysiological responses (Gerger et al., 2014). Thus, while negative emotions during aesthetic experiences may lead to pleasurable experiences in healthy individuals (Menninghaus et al., 2017), this may not necessarily be the case in AD patients with decreased ability to form abstractions. Hence, the relationship between liking of negative artworks and preference stability may differ between AD patients and healthy individuals: Whereas negative emotional valence of artworks might lead to higher preference (and preference stability) in healthy controls, in AD patients, negative valence of artworks may lead to less liking but higher affective processing of these images, thereby leading to higher stability of preferences for negative artworks in this group.

Furthermore, the severity of general cognitive impairment was not significantly related to AD patients' preference stability in any stimulus category in our study. This finding is consistent with previous results where no difference in preference stability between severity groups (mild, moderate, severe AD) was found (Graham et al., 2013). Thus, the role of disease severity for stability of preferences remains somewhat unclear, and future studies with bigger sample size may specifically study the relationship between severity of cognitive impairment and preference stability.

As it might be possible that stable preference rankings are obtained due to some artworks being generally preferred or disliked, we also analyzed the consistency of ranking positions for each individual stimulus. Average mean rankings as well as their standard deviations were relatively consistent with findings in Halpern et al. (2008), indicating a rather high disagreement between participants for stimuli of all stimulus categories. Moreover, higher arousal of artworks did not lead to higher agreement between participants which supports the idea of rather wide inter-individual differences in aesthetic preference ratings.

Interestingly, and contrary to our hypotheses, controls did not remember more positive and negative images compared to neutral stimuli. This may be due to a potential ceiling effect in this group as well as due to our stimuli not being of “extreme” emotional valence which may have led to rather vague differentiation between stimulus categories.

Given the nature of AD, the possibilities of the study design are limited. We strove for an optimal balance between gathering as much data as possible and simplifying the study design. Thus, the number of stimuli was limited. We still experienced concentration problems and motivational deficits in the AD group. Moreover, even though *VAPS*-images are rated on emotional valence by a quite high number of participants (*N* = 100), these ratings predominantly stem from younger and cognitively non-impaired participants who may perceive valence in art differently than participants in this study. Therefore, in the future, studies that allow to individually match the level of emotion for individual participants could be informative. Moreover, we did not measure the time spent on each preference ranking task. Hence, we cannot rule out that AD patients spent more time on average looking at negative images (compared to positive or neutral artworks) which may have contributed to our findings. However, we observed rather the opposite where participants, especially in the AD group, tended to dislike looking at the negative images which may have led to a *shorter* time spent on this task. Finally, the sample size is small, though comparable with earlier studies on aesthetic stability (Halpern et al., 2008; Graham et al., 2013), and our sample included more female (*n* = 10) than male participants (*n* = 5); generalizability of findings may therefore be limited.

Despite these limitations, this study provides promising avenues for future research and clinical practice, and revealed interesting preliminary findings on the role of emotional valence for the stability of aesthetic preferences in AD patients and healthy controls. The findings of our study support the idea of a somewhat preserved implicit emotional valence system in Alzheimer's dementia which has several implications for real-world applications. First, if there is such as an intact implicit valence system in AD patients, this is most relevant for everyone engaging with persons with dementia in daily life, both privately and professionally. Even though AD patients might not have explicit memory of certain encounters, they may still keep an emotional trace of these experiences, especially if these experiences are negative. Thus, exposing AD patients to any kind of negative experience should be avoided, and one should keep in mind that these experiences may negatively impact patients' life despite lacking explicit memory. Second, despite differences in stability of preferences, we observed that even persons with medium-severe levels of cognitive impairment were able to form subjective aesthetic judgments and indicate which artworks they preferred to other artworks. We therefore support the idea of including art therapy in AD patients' intervention plans. Focusing on those abilities that are still preserved in persons with dementia—such as meaningfully engaging with art—may help to balance out some of the adversities these individuals are faced with. Furthermore, we observed that displaying art to AD patients initiated some reflections on their own experiences (e.g., commenting on similarities between their living environment and the artwork), and participation in this study was positively evaluated throughout by all AD patients. We suggest that presenting AD patients more frequently with artworks, and even if it is just with a very simple reproduction of art like in this study, already helps to briefly relieve emotional distress in their daily life and may increase wellbeing and quality of life on a longer-term scale.

## Data Availability Statement

The raw data supporting the conclusions of this article will be made available by the authors, without undue reservation.

## Ethics Statement

This study involving human participants was reviewed and approved by Ethics Committee of the University of Vienna (Reference Number: 002800, 11/28/2017). The patients/participants provided their written informed consent to participate in this study.

## Author Contributions

EK: conceptualization, methodology, data collection, formal analysis, writing—original draft, review and editing, and data visualization. MF: planning and supervision of conceptualization, methodology, formal analysis, and worked on the first and final version of the manuscript. HL: planning and supervision of conceptualization and worked on the first and final version of the manuscript. All authors contributed to the article and approved the submitted version.

## Conflict of Interest

The authors declare that the research was conducted in the absence of any commercial or financial relationships that could be construed as a potential conflict of interest.

## Publisher's Note

All claims expressed in this article are solely those of the authors and do not necessarily represent those of their affiliated organizations, or those of the publisher, the editors and the reviewers. Any product that may be evaluated in this article, or claim that may be made by its manufacturer, is not guaranteed or endorsed by the publisher.
