# Supplementary material for: Aesthetic Preference for Negatively-Valenced Artworks Remains Stable in Pathological Aging: A Comparison Between Cognitively Impaired Patients With Alzheimer's Disease and Healthy Controls
Source: Front Psychol. 2022 May 26;13:879833. doi: 10.3389/fpsyg.2022.879833 (PMC9204348; doi:10.3389/fpsyg.2022.879833)
Supplement: Supplementary file 1 [file Data_Sheet_1.PDF]

Supplementary Figure 1. *Distributions of recognition scores shown per stimulus category and group*

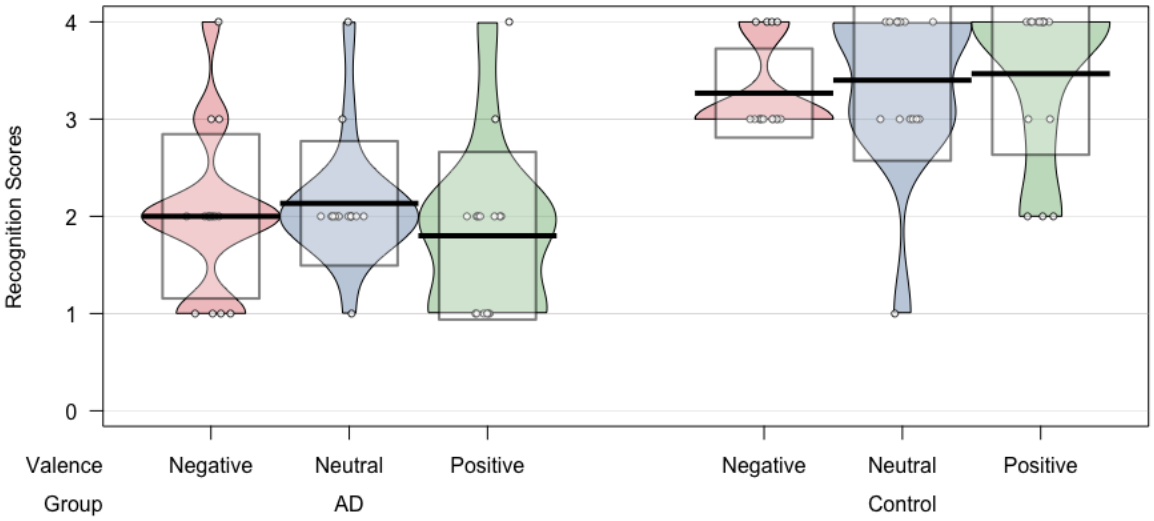

Note: Each dot represents the recognition score for one participant. Noise (jitter) was added horizontally to reduce overlap among points with similar value. Horizontal black lines indicate means. Standard deviation of the mean of recognition scores for each valence category are indicated as transparent boxes.

Supplementary Figure 2. *Frequency distribution of correct recognition scores (summed over all stimulus categories) per group.*

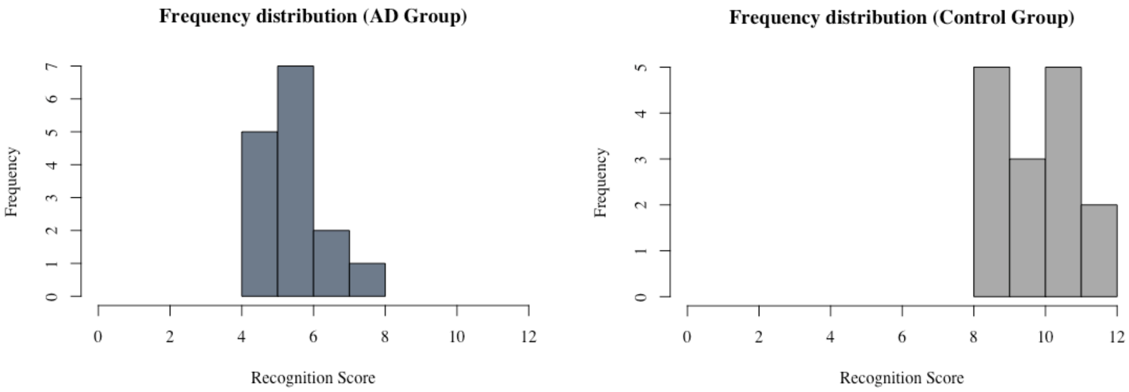

Note: The maximum of correct recognitions per person was 12 and consequently the recognition at chance was at six correct answers per person.
